# Market Dynamics and Economic Drivers of Poland’s Foreign Trade in Goose Meat and Offal

**DOI:** 10.3390/foods15081353

**Published:** 2026-04-13

**Authors:** Monika Wereńska, Wawrzyniec Michalczyk, Andrzej Okruszek

**Affiliations:** 1 Department of Food Technology and Nutrition, Wroclaw University of Economics and Business, 53-345 Wroclaw, Poland; andrzej.okruszek@ue.wroc.pl; 2 Department of International Economic Relations, Wroclaw University of Economics and Business, 53-345 Wroclaw, Poland; wawrzyniec.michalczyk@ue.wroc.pl

**Keywords:** goose meat, food system resilience, export specialization, consumer gap, niche markets

## Abstract

Poland ranks among the world’s leading exporters of goose meat and edible offal, yet domestic consumption remains minimal, revealing a structural imbalance between production and internal demand. This study aims to provide a comprehensive economic assessment of Poland’s foreign trade in goose meat and offal during 2020–2024, examining export specialization, price dynamics, and market resilience. Using official data from the Central Statistical Office (GUS), Eurostat, UN Comtrade, and the National Bank of Poland (NBP), trade flows were disaggregated by CN product codes, destination countries, and unit prices to identify key structural patterns. Results indicate that export volumes remained largely limited by price responsiveness despite sharp price increases and exchange rate fluctuations, confirming stable foreign demand. Exports were heavily concentrated in Germany, which absorbed over 70% of the total trade value, while domestic consumption stayed below 0.5 kg per capita annually. These findings demonstrate both the competitiveness and the fragility of Poland’s export-oriented trade model, characterized by dependence on a single market and limited domestic integration. The study concludes that long-term food system resilience requires diversification of export destinations, stimulation of domestic demand, and stronger alignment with sustainability goals. A forthcoming second part will address environmental impacts and consumer awareness.

## 1. Introduction

Poultry meat plays a significant role in the global agri-food sector, both as a staple protein source and as a tradable commodity [1]. Within this category, goose meat is a niche product with high gastronomic and cultural value in selected regions. It is particularly appreciated in countries such as Germany, Austria, France, China, and Egypt. Although global production of goose meat is relatively small compared to other poultry types, its economic and symbolic importance in specific markets justifies focused scientific attention [2,3]. Poland, as one of the world’s largest producers and exporters of goose meat and edible offal, stands at the forefront of this specialized trade.

The Polish goose industry is dominated by the White Kołuda^®^ goose, commonly referred to as the “Polish oat goose” due to the final oat-based fattening phase, which is believed to enhance sensory characteristics and improve the fatty acid profile. This production method results in meat with a high content of unsaturated fatty acids and favorable health lipid indices. Approximately 95% of Poland’s goose meat production is exported, with Germany accounting for the vast majority of export volume. This reflects a unique market configuration where the domestic consumer base remains underdeveloped, and international markets drive industry growth [4,5]. Despite its strong export orientation, goose meat consumption in Poland is marginal -estimated at less than 0.5 kg per capita per year. Several factors contribute to this phenomenon, including price sensitivity, lack of culinary tradition among younger generations, and limited availability in retail chains. While various promotional campaigns have been launched to revive interest in goose products domestically, their effects have been limited. As a result, Polish producers remain highly dependent on foreign demand, making the sector vulnerable to international market disruptions and policy changes.

The years 2020–2024 have brought a series of shocks and structural shifts to international agri-food trade. The COVID-19 pandemic disrupted logistics and food service sectors, while subsequent inflationary pressures and exchange rate volatility affected pricing strategies and consumer behavior. In parallel, geopolitical tensions, particularly in Eastern Europe, influenced trade policy and regional cooperation. These factors have altered the economic landscape in which Polish goose producers operate, necessitating a reevaluation of trade patterns, price trends, and the strategic resilience of the food system [6].

Although goose meat constitutes a niche product within the global poultry sector, Poland holds a unique position as one of its leading producers and exporters. Despite this strategic status, no comprehensive study has yet provided a systematic, multi-year analysis of Poland’s foreign trade in goose meat and edible offal, including both export and import structures, product categories, destination/origin countries, price indices, and exchange rate effects. Existing literature tends to focus on biological, technological, or nutritional aspects of goose production [5,7,8,9,10,11,12,13,14,15,16,17,18,19,20,21,22], while the economic and trade dimensions remain significantly underexplored.

The aim of this study is therefore to fill this knowledge gap by providing a detailed and disaggregated economic analysis of Poland’s foreign trade in goose meat and offal over the 2020–2024 period. The study synthesizes data from official statistical sources—Główny Urząd Statystyczny (GUS, Central Statistical Office) [23], Narodowy Bank Polski (NBP, National Bank of Poland) [24], Eurostat [25], UN Comtrade [26], and evaluates trade flows by product type, trade partner, price trends, and macroeconomic context. Special attention is paid to trade concentration, market resilience, and the implications for food policy and sustainable animal production systems.

From an economic standpoint, such an analysis is crucial, although small in volume, the goose meat sector generates significant export value and exemplifies a highly specialized value chain with strong export orientation but limited domestic integration. Understanding its trade dynamics offers valuable insights for policymakers, producers, and researchers interested in niche meat markets, regional agri-food specialization, and food system resilience.

Beyond quantitative trade analysis, the study examines structural aspects of the Polish goose sector, including product specialization, export concentration, and the limited development of the domestic market. The findings are interpreted in relation to broader themes in food policy, such as economic resilience, export dependency, and the role of niche animal products in sustainable food systems. The paper also explores potential development scenarios, including diversification of export destinations, greater value addition through product innovation, and the integration of goose meat into clean-label and functional food trends.

The main objective of this study is to provide a detailed economic analysis of Poland’s foreign trade in goose meat and edible offal over the period 2020–2024.

The analysis follows an integrated descriptive framework wherein export concentration patterns (Section 3.1, Section 3.2 and Section 3.3) are examined alongside product specialization dynamics (Section 3.4, Section 3.5, Section 3.6, Section 3.7 and Section 3.8), price transmission mechanisms), and consumer-level demand constraints (Section 3.14). This structure enables identification of structural interdependencies: high geographic concentration limits negotiating power and creates demand-shock vulnerability; the absence of domestic demand removes a natural absorption buffer for production fluctuations; and consumer barriers perpetuate export dependence.

This study positions itself as a descriptive trade diagnostic analysis, providing the first systematic, disaggregated empirical documentation of Poland’s goose meat trade patterns, a sector for which no comparable multi-year analysis exists in the English-language literature. The contribution lies in mapping structural features, identifying interdependencies, and establishing an empirical foundation for future analytical work, rather than in econometric identification of causal mechanisms.

## 2. Materials and Methods

### 2.1. Research Objectives and Hypothesis

The overarching objective of this study is to provide a comprehensive economic analysis of Poland’s foreign trade in goose meat and edible offal over the period 2020–2024. Particular attention is paid to structural trade characteristics, market resilience, domestic consumption patterns, and emerging sustainability implications. The study focuses on the following specific aims:

- to identify the structural features of Poland’s export specialization in goose meat;
- to evaluate the resilience of this sector to macroeconomic disturbances, particularly exchange rate fluctuations;
- to assess the degree of market concentration and dependence on individual trading partners;
- to investigate the persistent gap between high export performance and low domestic consumption;
- to provide a preliminary outlook on the sustainability implications of Poland’s current export-driven trade model.

Based on these objectives, the following research hypotheses were formulated:

**H1:**  *The volume of Poland’s goose meat exports exhibits limited responsiveness to changes in the EUR/PLN exchange rate, suggesting that demand is driven primarily by product specialization rather than price competitiveness*.

**H2:**  *Poland’s goose meat exports are geographically concentrated, with a strong reliance on the German market, reducing the sector’s strategic resilience*.

**H3:**  *Despite its global export leadership, Poland continues to record structurally low domestic consumption of goose meat, due to cultural, economic, and informational barriers*.

These hypotheses are tested using a combination of descriptive statistical analyses, disaggregated trade data, and original consumer survey results. Findings are contextualized within broader food policy and sustainability frameworks. 

Given the number of comparisons conducted, the possibility that some individually significant results (particularly those near the conventional α = 0.05 threshold) may reflect chance variation should be acknowledged. No formal multiplicity adjustment (e.g., Bonferroni correction) was applied, as the survey component is exploratory in nature; accordingly, marginal results should be interpreted with appropriate caution.

### 2.2. Data Sources

The quantitative analysis is based on secondary data compiled from a range of authoritative and internationally recognized sources: The Central Statistical Office of Poland (GUS) provided detailed statistics on domestic production, exports, and imports of goose meat and offal, Eurostat and the United Nations Comtrade Database facilitated international comparisons and validation of trade flows by product category and trading partner, The National Bank of Poland (NBP) served as the source of official EUR/PLN exchange rates, allowing for currency-adjusted price analysis. All data were cross-validated where applicable to ensure consistency and accuracy.

### 2.3. Product Classification

Trade analysis was based on product categories defined by the Combined Nomenclature (CN) according to Commission Implementing Regulation (EU) 2023/2364 of 26 September 2023 [27], specifically group 02075, which covers various types of goose meat and edible offal. To account for the heterogeneous nature of certain categories, a refined classification into six homogeneous groups was adopted:

Meat, not cut in pieces, fresh or chilled—CN codes: 0207 51 10, 0207 51 90.

- Meat, not cut in pieces, frozen—CN codes: 0207 52 10, 0207 52 90.
- Meat in cuts, fresh or chilled—CN codes: 0207 54 10, 0207 54 21, 0207 54 31, 0207 54 41, 0207 54 51, 0207 54 61, 0207 54 71, 0207 54 81.
- Meat in cuts, frozen—CN codes: 0207 55 10, 0207 55 21, 0207 55 31, 0207 55 41, 0207 55 51, 0207 55 61, 0207 55 71, 0207 55 81.
- Edible offal, fresh or chilled—CN codes: 0207 53 00, 0207 54 91, 0207 54 99.
- Edible offal, frozen—CN codes: 0207 55 93, 0207 55 95, 0207 55 99.

This level of disaggregation enabled the identification of product-specific trade dynamics, including pricing mechanisms, specialization trends, and partner country preferences. For instance, high-value products such as fatty goose livers (CN 0207 53 00) generated disproportionately high export revenues despite low volumes, while frozen products formed the bulk of mass-market exports, particularly to Germany and France.

### 2.4. Study Period and Scope

The study encompasses the years 2020–2024, allowing for the observation of trade patterns before, during, and after the COVID-19 pandemic. This timeframe provides a relevant context for analyzing the resilience of the goose meat trade to exogenous shocks and structural changes in demand.

### 2.5. Data Processing and Analytical Approach

Data processing and analytical approach. Descriptive statistical methods, including trend analysis and concentration ratios, were employed to evaluate market structure, stability, and partner dependence. Trade flows were examined by volume (kg), value (EUR and PLN), and unit price (EUR/kg and PLN/kg). Time series were constructed to analyze trends in export value, volume, and pricing. Price indices were adjusted for exchange rate movements. To assess the effect of exchange rate fluctuations, EUR- and PLN-denominated price indices were compared across product groups and time.

### 2.6. Consumer Survey

To complement the statistical analysis and gain deeper insights into domestic consumption trends, an original consumer survey was conducted annually between 2022 and 2025. The survey aimed to explore consumer attitudes toward goose meat, identify key consumption barriers, and assess public awareness of Polish goose products.

The data collection instrument was a structured questionnaire comprising 22 items, including both single-choice and multiple-choice questions. One semi-open question allowed respondents to provide qualitative comments. Items 1–5 captured sociodemographic characteristics (age, gender, education, type of residence, and region), while items 6–22 focused on consumer behavior and perceptions related to goose meat, including:

- frequency and context of consumption;
- awareness of regional goose products;
- perceived consumption barriers (e.g., high price, limited availability, lack of culinary familiarity);
- willingness to pay and perceived nutritional value;
- awareness and perceived effectiveness of promotional campaigns.

The survey was distributed online using the CAWI method (Computer-Assisted Web Interviewing), implemented via Microsoft Teams Forms. In-person administration was limited due to COVID-19 restrictions and public health considerations. The consumer survey included in this study was conducted with the approval of the Ethics Committee of Wroclaw University of Economics and Business (Ex post approval No. 08/2026). Applications for ethical approval were submitted as part of the grant project, covering the entire scope of the research, including the consumer survey. All participants provided informed consent prior to completing the survey. Participation was voluntary, and respondents were informed about the purpose of the study, their right to withdraw at any time, and the anonymous nature of the survey. All participants provided informed consent prior to completing the survey. Before starting the questionnaire, participants were informed that: the purpose of the survey is for research within the grant project; the collected data will be used in scientific analyses and publications; participation is voluntary, and they can withdraw at any time without any consequences; the survey is fully anonymous and no personal or sensitive data are collected; by continuing with the survey, they confirm that they understand and agree with these conditions.

A total of 1.036 (N = 1.036) valid responses were collected over the four-year period, with annual distributions as follows: 2022 (260), 2023 (248), 2024 (272), and 2025 (256). Since no statistically significant year-to-year variation was observed across the four editions of the survey (2022–2025), the results presented below are based on aggregate averages calculated from the full sample (N = 1.036). This approach allowed for a more robust representation of general consumer attitudes and perceptions, without loss of analytical clarity. Respondents were adult residents of Poland aged 18 and over, with representation across gender, educational backgrounds, and regions.

Survey responses were analyzed using descriptive statistics and cross-tabulations. To provide a synthesized view, arithmetic means of selected quantitative indicators were calculated across the four-year period (2022–2025), yielding a representative profile of consumer attitudes and perceptions. This averaging approach, while smoothing year-to-year variability, allowed for the identification of persistent structural patterns. Where relevant, year-specific divergences are noted and interpreted contextually. Particular analytical focus was placed on sociodemographic differentials, especially age, gender, education level, and region, to explore how these factors shaped attitudes toward goose meat consumption. The survey results were integrated with secondary trade and consumption data to enhance understanding of the structural disconnect between Poland’s strong export orientation and persistently weak domestic demand. The survey component was guided by the following general hypothesis: H_0_: Consumer knowledge about goose meat in Poland is insufficient.

Survey data were collected from adult Polish residents using a CAWI approach (Computer-Assisted Web Interviewing) over the period 2022–2025 (total N = 1.036). Because the sampling frame was non-probability, descriptive statistics were primarily reported, and where appropriate, model-based inference with robust standard errors was applied. Frequencies and percentages were calculated to summarize all survey responses, and bar charts were used to visually present the distributions. Continuous variables, such as age, were summarized with means and standard deviations (SD). We acknowledge that the results are not fully generalizable to all Polish consumers, as over 50% of survey respondents were drawn from cities with more than 500,000 inhabitants.

Differences in categorical survey responses between women and men, or between other sociodemographic groups, were assessed using Fisher’s exact tests, especially when some response categories had small counts. Each response option in multiple-choice questions was treated as a separate binary variable (selected/not selected). The resulting *p*-values indicate whether the proportion of participants choosing a specific response differs significantly by group. All statistical analyses were conducted using Statistica software version 13.3.

Descriptive statistics were used to summarize the demographic characteristics of the participants. Categorical variables, such as sex and age group, are presented as frequencies and percentages. Age was treated as a continuous variable, with midpoint values assigned to each age group (18–30: 24 years; 31–50: 40.5 years; >50: 60 years) to calculate mean age and standard deviation (SD) using a weighted approach. Differences in age between sexes were assessed using one-way analysis of variance (ANOVA). All statistical analyses were performed using Statistica software, with a significance level set at α = 0.05.

## 3. Results

### 3.1. Export Value of Goose Meat and Offal from Poland in 2015–2024

**Figure 1:**
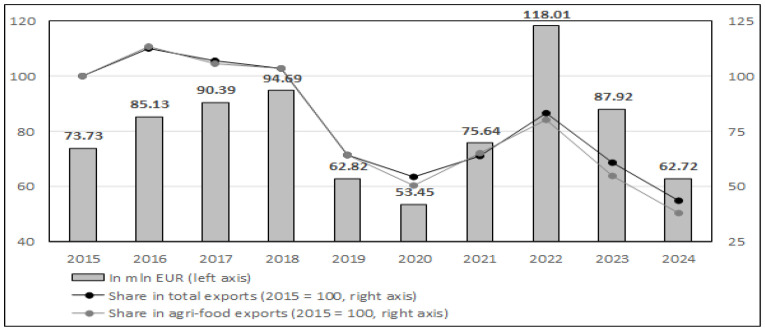
Polish exports of meat and edible offal of geese in the years 2015–2024. Source: own development based on [23].

Figure 1 presents the annual value of Poland’s exports of goose meat and edible offal in the period from 2015 to 2024, based on data from the Central Statistical Office of Poland (GUS). Poland’s exports of goose meat and edible offal demonstrated significant volatility over the analyzed period, with values increasing from €53.45 million in 2020 to a peak of €118.0 million in 2022, followed by a sharp decline to €62.72 million in 2024. This represents a 121% increase from 2020 to the 2022 peak, followed by a 47% contraction by 2024. The peak in 2022 coincided with multiple concurrent market disruptions, including post-pandemic inflationary pressures, the outbreak of highly pathogenic avian influenza across Europe, and the Russia-Ukraine conflict’s impact on feed costs.

The subsequent decline in 2023–2024 suggests market normalization following extraordinary price conditions rather than structural erosion of export capacity. Nevertheless, the pronounced amplitude of these fluctuations underscores the sector’s vulnerability to external macroeconomic and biological transformation.

Table 1 reveals an exceptionally concentrated export structure. Germany dominated throughout 2020–2024, absorbing 74–86% of total export value and yielding a Herfindahl-Hirschman Index exceeding 5500 more than double the threshold for highly concentrated markets (own calculations based on data, using explanation defined by Eurostat on page [28]. Secondary European markets (France 4–7%, UK 1–2.5%, Austria, Czechia) provided limited diversification. Non-European destinations exhibited high volatility: Hong Kong’s share collapsed from 8.2% (2020) to 0.8% (2022) before recovering to 5.7% (2024), illustrating both expansion potential and demand instability in distant markets. Notably, concentration intensified during the 2022 price peak rather than triggering diversification, confirming that demand reflects product differentiation rather than price sensitivity.

### 3.2. Import Volume of Goose Meat and Offal from POLAND in 2015–2024

**Figure 2:**
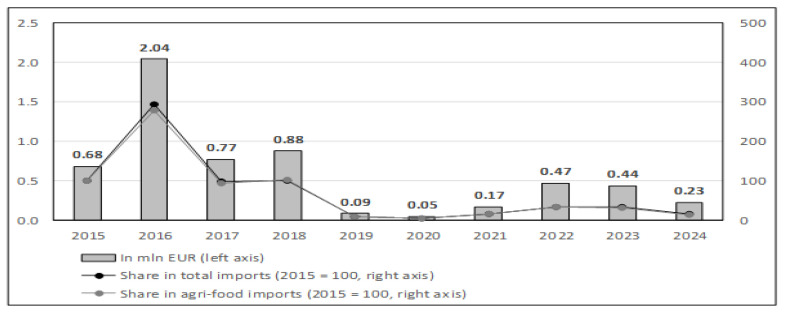
Polish imports of meat and edible offal of geese in the years 2015–2024. Source: own development based on [23].

Figure 2 illustrates the annual import volume of goose meat and edible offal into Poland between 2015 and 2024. The data reveal a relatively stable supply of these products, with volumes ranging from 15,432 to 16,980 tons per year. Polish imports of goose meat and offal, measured in hundreds of thousands of EUR annually, constitute only a marginal fraction of the export value of this product group, remaining below 0.5% since 2019. These imports also display considerable fluctuations, both in absolute EUR terms and as a proportion of total Polish imports and agri-food imports. Notably, the directional trends in recent years largely mirror those observed in exports, suggesting a potential interdependence between the two flows or common underlying market factors such as pricing dynamics or seasonal demand.

This decoupling between value and volume indicates that, despite variability in unit prices, driven by factors such as inflation, exchange rates, and input costs, the demand for imported goose meat in Poland has remained relatively stable in physical terms. The persistent but limited import activity may reflect niche domestic demand unmet by local production or the seasonal preferences of certain consumer segments. However, continued reliance on external suppliers, even at low volumes, underscores the importance of maintaining resilient supply chains and balancing imports with sustainable domestic production strategies in alignment with broader food security goals.

**Figure 3:**
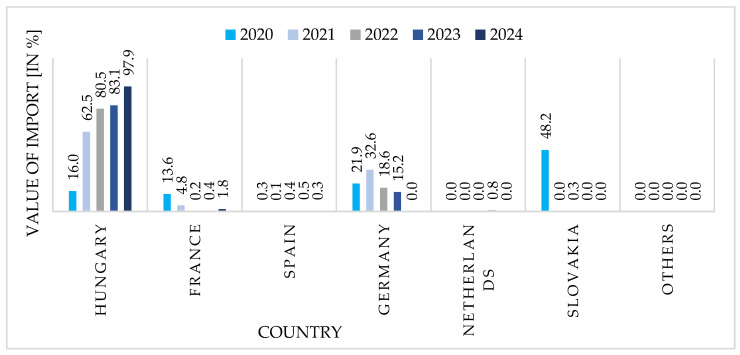
Polish imports of meat and edible offal of geese in the years 2020–2024, by country of origin share (in %). Source: own development based on [23].

Figure 3 documents a rapid consolidation of import sources. Hungary’s share surged from 16% (2020) to 98% (2024), effectively monopolizing Poland’s goose meat imports as Germany (22% → 0%) and Slovakia (48% → 0%) exited entirely. This transformation reflects competitive pricing advantages and regional supply chain integration, but creates significant vulnerability: near-total dependence on a single supplier exposes the sector to disease outbreaks, trade disruptions, or policy shifts in Hungary. Balancing logistical efficiency against supply security will require either cultivating alternative partnerships or expanding domestic processing capacity.

### 3.3. Polish Foreign Trade in Meat and Edible Offal

**Figure 4:**
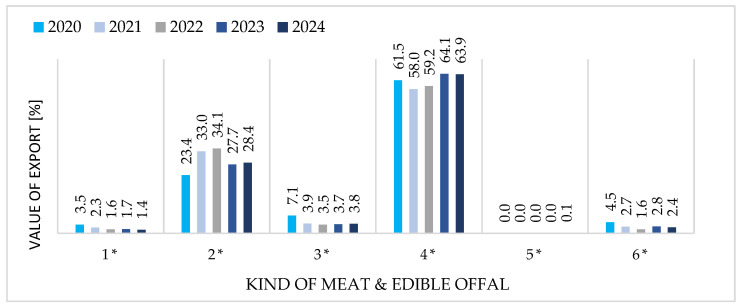
Polish foreign trade exports in meat and edible offal of geese in the years 2020–2024, by product group share (in %). Explanation: 1 * Meat, not cut in pieces, fresh or chilled; 2 * Meat, not cut in pieces, frozen; 3 * Meat, in cuts, fresh or chilled; 4 * Meat, in cuts, frozen; 5 * Edible offal, fresh or chilled; 6 * Edible offal, frozen. Source: own development based on [23].

**Figure 5:**
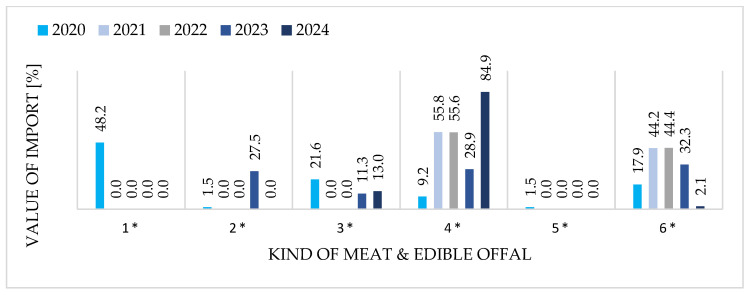
Polish foreign trade imports in meat and edible offal of geese in the years 2020–2024, by product group share (in %). Explanation: 1 * Meat, not cut in pieces, fresh or chilled; 2 * Meat, not cut in pieces, frozen; 3 * Meat, in cuts, fresh or chilled; 4 * Meat, in cuts, frozen; 5 * Edible offal, fresh or chilled; 6 * Edible offal, frozen. Source: own development based on [23].

Figure 4 and Figure 5 provide a comparative overview of the structure of Polish exports and imports of goose meat and edible offal, disaggregated into six product groups and expressed as percentage shares of total trade value for each year from 2020 to 2024.

Figure 4 and Figure 5 reveal a pronounced structural asymmetry. Exports are highly concentrated in two frozen product categories: cuts (60–65% of value) and whole carcasses (30–35%), reflecting Poland’s processing capacity and alignment with German market preferences. Fresh products and offal remain marginal (<5% combined), constrained by perishability and logistics. Import structure, by contrast, exhibits high interannual volatility characteristic of opportunistic sourcing rather than strategic supply management. This asymmetry confirms Poland’s position as a specialized, vertically integrated exporter while underscoring the peripheral role of imports in the domestic market.

### 3.4. Export Side—Strong Specialization in Frozen Cuts & Import Side—Structural Evolution and Volatility

Export specialization remained stable throughout 2020–2024: frozen cuts dominated (58–64%), followed by frozen whole carcasses (23–34%), while fresh products (<8%) and offal (<5%) remained marginal due to perishability constraints and domestic by-product absorption. Import structure, conversely, underwent consolidation—shifting from a diversified 2020 portfolio (fresh carcasses from Slovakia, cuts from Germany/France, offal from Hungary) to near-exclusive reliance on Hungarian frozen cuts by 2024. This parallel concentration in both product categories and supplier base reflects regional supply chain optimization but amplifies systemic risk.

Table 2 provides a detailed breakdown of the value of Poland’s exports of goose meat and edible offal across five major product groups and multiple destination countries between 2020 and 2024. The data are disaggregated by product category in accordance with Combined Nomenclature (CN) codes and by importing country, revealing both product specialization and market concentration trends.

### 3.5. Export Structure by Product Group

The majority of Polish goose exports consisted of frozen meat in cuts, which accounted for the largest share of total export value, reaching €69.9 million in 2022 and €40.1 million in 2024. This reflects Poland’s specialization in value-added, semi-processed products with extended shelf life and high transport stability. Germany remained the principal importer in this category, consistently purchasing over 75% of frozen cuts each year. Other significant product groups included frozen whole carcasses (CN 0207 52 90) and fresh cuts (CN 0207 54 xx), but these played a secondary role in export value. The share of fresh or chilled, uncut carcasses was minimal and declining (from €1.9 million in 2020 to €0.9 million in 2024), mainly due to perishability and logistical limitations in distant markets.

### 3.6. Geographic Concentration of Exports

Germany was by far the most important destination across all product categories, especially in frozen segments. For example:

- It absorbed over 80% of exports of frozen whole carcasses and frozen cuts;
- In some years, e.g., 2022, Germany alone accounted for over €61 million worth of Polish goose meat.

Other European countries, such as France, Austria, Czechia, and the UK, played a modest but stable role in import demand. Interestingly, Hong Kong and Canada appeared as non-European markets for frozen cuts and offal, though with limited overall contribution (under 5%).

### 3.7. Temporal Trends and Shifts

The total export value peaked in 2022 at €118 million, before declining to €62.7 million in 2024. This fall was most visible in Germany, which reduced its import volume and value from 2022 onward. Nonetheless, some smaller markets like France and the UK showed a steady increase, indicating opportunities for diversification.

### 3.8. Policy and Trade Implications

The documented export monostructure, frozen cuts to Germany, creates systemic vulnerabilities that emerging markets (Canada, Hong Kong) cannot yet offset. Three strategic priorities emerge: product innovation toward higher-margin convenience formats, systematic cultivation of non-EU markets through geographical indication branding, and domestic market development to provide demand-shock absorption capacity. Without diversification, the sector remains exposed to German demand fluctuations and policy shifts.

Table 3 presents the detailed structure of Poland’s imports of goose meat and edible offal over the five-year period from 2020 to 2024. The data are disaggregated by product group—aligned with Combined Nomenclature (CN) classification and by country of origin, offering a comprehensive view of the evolution of import patterns in terms of both product diversity and trade partner concentration.

### 3.9. Structural Reconfiguration of Import Flow

A remarkable finding is the profound transformation of the product mix and origin countries during the study period. In 2020, the import portfolio was relatively diversified, comprising:

- Fresh whole carcasses (CN 0207 51) from Slovakia (€22.6 k);
- Fresh cuts from Germany and France (€5.2 k and €4.9 k respectively);
- Frozen offal primarily from Hungary (€7.5 k).

However, by 2024, imports had become heavily concentrated in two segments:

- Frozen meat in cuts (CN 0207 55), amounting to €191.4 k (all from Hungary);
- Fresh cuts (€29.4 k), again almost exclusively from Hungary.

This transition reflects both a narrowing of the product base and a strategic realignment of sourcing partners, increasingly favoring intra-EU trade with Hungary.

### 3.10. Dominance of Hungary as Sole Supplier

Hungary’s role as Poland’s primary supplier of goose meat became nearly absolute by 2024. In the frozen meat category, Hungary’s share rose from 0% in 2020 to 100% in 2024, while Germany, once a major partner, phased out entirely. Similarly, Hungary became the exclusive source of fresh cuts in 2024, replacing earlier multi-country sourcing patterns. This concentration suggests long-term bilateral trade agreements or the consolidation of supply chains driven by logistics, veterinary standards, and competitive pricing. Yet, this model raises concerns about supply chain resilience, especially in the event of disease outbreaks or export restrictions in Hungary. This concern is compounded by the fact that Hungary itself accounted for 24% of all avian influenza outbreaks in EU poultry during the 2021–2022 epidemic wave [29], making Poland’s near-exclusive import source simultaneously one of Europe’s most disease-exposed goose producers. Moreover, Hungarian goose production has exhibited long-term structural instability, output declined by approximately 45% between 2003 and 2013, driven in part by animal welfare campaigns and sector restructuring, suggesting that dependence on a single, historically volatile supplier amplifies the systemic risk identified above [30].

### 3.11. Volatility in Offal Imports

Import values of frozen offal (CN 0207 55 99) peaked in 2022 at €209.5 k, dominated by Hungary, but fell sharply to €4.7 k in 2024. This contraction may reflect a combination of reduced demand, saturated domestic processing capacities, or re-export diversion. The near disappearance of French and Spanish offal imports further underscores the volatility and short-term opportunism in offal sourcing.

### 3.12. Decline in Imports of Fresh Whole Carcasses

The complete cessation of imports of fresh, whole goose carcasses after 2020, once sourced from Slovakia, reveals a significant structural change. Possible reasons include reduced domestic demand, logistical challenges (shelf-life), or shifting consumer preferences toward processed or portioned products. The data indicate a trend away from raw carcass imports in favor of semi-processed cuts.

### 3.13. Strategic and Sustainability Implications

The data in Table 3 underscore a growing reliance on a single partner (Hungary) and two product categories (frozen and fresh cuts), suggesting systemic risks in Poland’s goose meat import model. While such consolidation may reduce transaction costs and improve traceability, it also limits Poland’s strategic flexibility and may exacerbate price volatility or supply shocks. From a sustainability perspective, the dominance of frozen products facilitates lower spoilage and longer shelf-life, which aligns with food waste reduction goals. However, it may also increase the energy footprint (e.g., cold chain requirements), meriting further life cycle assessment. To build a more resilient and sustainable import structure, policy recommendations include:

- Encouraging product and partner diversification;
- Strengthening domestic processing to reduce import needs;
- Enhancing bilateral trade diplomacy with underutilized sources like France or Spain;
- Monitoring market dependencies via early-warning systems.

A more detailed breakdown of product categories, specifically those assigned an eight-digit code under the Combined Nomenclature, allows for the identification of the key Polish export items (Table 4). The leading products include:

- Frozen whole goose carcasses, commonly referred to as “75% geese” (CN 0207 52 90: “meat, not cut in pieces, frozen: plucked and drawn, without heads and feet”), with annual export values ranging from 13 to 40 million EUR (average: 24 million EUR) and volumes between 5 and 7 million kg per year,
- Frozen goose legs with bone (CN 0207 55 61: “meat, in cuts, frozen, with bone: legs”), with an export value fluctuating between 17 and 33 million EUR annually (average: 24 million EUR), and volumes of approximately 3 to 4 million kg per year,
- Frozen goose breasts with bone (CN 0207 55 51: “meat, in cuts, frozen, with bone: breasts”), exported at values between EUR 9 and 28 million annually (average: 16 million EUR), with a yearly volume of around 2 million kg.

Exports of all other CN product groups remain below 10 million EUR annually.

In contrast, identifying the most significant import categories proves more challenging due to substantial year-to-year fluctuations and the absence of any CN product group with uninterrupted trade flows throughout the observed period; each recorded at least one year without transactions (Table 5). However, based on the arithmetic average of annual import values between 2020 and 2024, “edible offal, frozen: other than livers” (CN 0207 55 99) emerges as the dominant category, with an average annual value of 85 thousand EUR and an average volume of 45 tonnes. The next most significant product is “meat in cuts, frozen, with bone: other than halves, quarters, whole wings, backs, necks, rumps, wing-tips, breasts, legs or paletots” (CN 0207 55 81), with average annual imports of approximately 40 thousand EUR and a volume of 16 tonnes. All remaining CN product groups show much lower average annual import values (27 thousand EUR or less).

**Figure 6:**
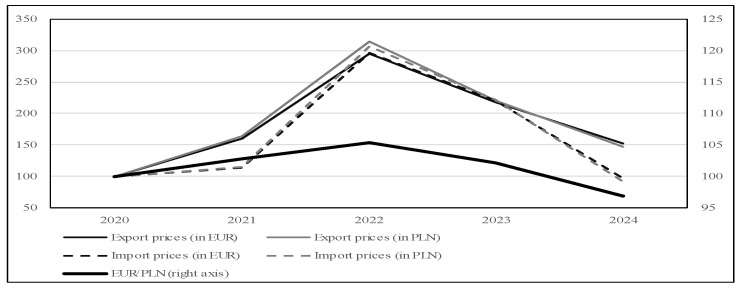
Average prices per kilogram in Polish foreign trade in meat and edible offal of geese and average EUR/PLN exchange rate in the years 2020–2024 (2020 = 100).

An analysis of changes in the prices of exported and imported goods expressed in EUR and PLN per kilogram reveals a marked increase in 2022 (Figure 6). The surge is substantial-export prices in euros almost doubled compared to the previous year (an increase of about 85%), while import prices even nearly tripled (rising by approximately 160%) compared to the base year 2020. By 2024, the prices had returned to levels observed in 2020–2021. These fluctuations can be attributed in part to exchange rate volatility between the euro and the Polish zloty; however, this factor accounts for only a minor portion of the observed changes. The EUR/PLN exchange rate rose in 2022 by about 5% compared to 2020, followed by a decrease of approximately only 8% by 2024.

The price increase in 2022 was particularly evident for the key Polish export items (Table 6). Prices of frozen “75% geese” (CN 0207 52 90) and frozen goose legs with bone (CN 0207 55 61), expressed in EUR, rose by around 80% compared to the previous year, while prices of frozen goose breasts with bone (CN 0207 55 51) increased by more than 100%. A significant price rise of over 85% was also recorded for the CN product group “meat in cuts, frozen, with bone: other than halves, quarters, whole wings, backs, necks, rumps, wing-tips, breasts, legs or paletots” (CN 0207 55 81). In contrast, conducting a comparable analysis for imports is challenging, as the importation of individual CN product groups does not occur every year. Nevertheless, for instance, prices of frozen goose legs with bone (CN 0207 55 61) increased by nearly 170% over the same period, those of “edible offal, frozen: other than livers” (CN 0207 55 99) rose by more than 90%, and prices for other frozen meat cuts with bone (CN 0207 55 81), not shown in Table 6 due to the absence of imports in 2020, tripled.

Shifting to a more general level of reflection, it is important to note that Poland is the leading exporter of meat and edible offal of geese within the European Union, with only Hungary exhibiting comparable export values. In 2023, Hungarian exports amounted to 78 million EUR, followed by 62 millionEUR in 2024. In contrast, exports from other EU member states did not exceed 5 mln EUR annually [25]. When taking into account the data for the whole world, Poland also ranks among the top exporters, alongside China and Hungary. According to UN data, only these three countries reported export values of meat and edible offal of geese exceeding 80 million USD in 2023, while all other countries exported less than 4 million USD that year [26]. It is also worth noting that China accounts for approximately 95% of the world’s goose meat production [31].

In the case of Poland, the main reason for such large export volumes is the relatively very low prices. Goose meat produced in Poland can be cheaper than German goose meat as the Polish goose is produced within an integrated, large-scale export-oriented system characterized by low labour and feed costs, whereas the German product comes from smaller-scale, premium and often organic or traditional production systems. In this context, it should be noted that the significant increase in goose meat prices in 2022 was caused by the loss of a large part of the flock due to avian influenza, as well as by very high feed prices; compared to 2021, there was an increase of more than one-third. Avian influenza in Europe predominantly affected France (68% of the overall outbreaks in poultry), but also Hungary, Poland’s main competitor (24% of outbreaks) [29]. This transitional decline in supply is concomitant with a continuous and steady increase in demand for goose meat, driven by its growing consumption worldwide over many years and by the change in consumer preferences. This change primarily stems from the pursuit of more sophisticated, demanding and unconventional products, such as goose meat, as well as from the relatively short production cycle of goose meat and its cost-effectiveness.

However, despite Poland’s dominant position in the global market, domestic consumption of goose meat remains relatively low, especially due to the lack of culinary tradition involving this type of poultry. Although per capita consumption has more than doubled over the past decade, primarily as a result of many national promotional campaigns, it still does not exceed 0.3 kg per person annually [32]. Consequently, producers are compelled to seek foreign markets for their products. In this context, it is worth noting that the aggregate trade volume, along with the volumes of individual product groups, appears to exhibit limited sensitivity to fluctuations in the exchange rate.

The depreciation of the Polish zloty against the euro between 2020 and 2022 was not accompanied by an increase in total export volume; on the contrary, a decline was recorded. Likewise, the subsequent appreciation of the currency in 2023 and 2024 did not result in a reduction in export quantities measured in kilograms. Import volumes similarly showed no discernible correlation with movements in the zloty-euro exchange rate. Notably, total imports followed a consistently upward trajectory, irrespective of exchange rate dynamics. The authorities support Polish producers through investments in promotional campaigns that highlight Polish goose meat not only domestically, but also internationally. To date, two campaigns have been conducted abroad using its resources: on the German market since 2021, titled “Gans und Ente aus Polen. Probieren Sie, was Ihr Nachbar zu bieten hat!” (“Goose and duck from Poland. Try what your neighbour has to offer!”) [33] and in the Czech Republic, starting in 2024, called “Kachna a Husa z Polska. Pokud je něco dobrého, obhájí se to” (“Duck and goose from Poland. If something is good, it will defend itself”) [34].

**Figure 7:**
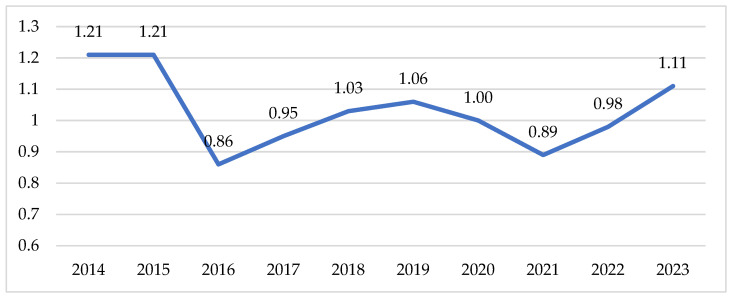
Goose population in Poland in the years 2014–2023 (as of December, in millions of birds). Source: own development based on [23].

In recent years, the population of geese in Polish farms has remained stable, fluctuating between 0.85 and 1.25 million birds (Figure 7). The dominant breed, accounting for 90–95% of the total stock, is the White Koluda Goose (commonly referred to as the “oat goose”). This breed is the result of long-term research and selection efforts conducted at the Experimental Station of the National Research Institute of Animal Production in Koluda Wielka. It has been officially included in the List of Traditional Products by the Ministry of Agriculture and Rural Development of the Republic of Poland on 10 August 2023 and recognized for the exceptional sensory characteristics of its meat (including a pleasant fat aroma). Moreover, the breed demonstrates favourable pre-slaughter body weight, efficient feed conversion, good health status and adaptability to diverse environmental conditions. These distinctive features of this goose breed and of the meat derived from it contribute to its exceptionally strong competitive position in international markets [35].

### 3.14. Linking Consumer Behavior to Trade Patterns

The trade analysis presented in the preceding sections identifies a pronounced imbalance between Poland’s strong export performance and its very low level of domestic consumption, estimated at below 0.3 kg per capita annually. While trade statistics clearly document the extent of export orientation, approximately 95% of total production, they do not, by themselves, explain why domestic demand remains limited. Addressing this issue requires consideration of consumer-level factors.

The consumer survey was therefore designed to complement the trade analysis by providing micro-level insights into consumption-related constraints. From a food consumer behavior perspective, the survey aims to identify economic, perceptual, and experiential factors that may inhibit domestic consumption. In this context, limited domestic demand can be understood as a demand-side condition that contributes to high export dependence, rather than as an outcome of production limitations.

Survey results indicate that low consumption levels are associated with high price sensitivity relative to more familiar food alternatives, limited culinary familiarity and confidence in preparation, and restricted retail availability. These factors help contextualize the trade patterns discussed in Section 3.13, where export dominance coincides with minimal domestic market penetration. The persistence of such consumer-level barriers may help explain why increased production capacity has not translated into greater domestic uptake.

By explicitly linking consumer behavior with aggregate trade outcomes, this section provides a conceptual bridge between micro-level consumption patterns and macro-level market structure. The findings suggest that the sector’s reliance on export markets is shaped not only by international competitiveness but also by demand-side constraints within the domestic market. From the perspective of food consumer research, addressing these constraints may be relevant for strategies aimed at supporting more balanced market development.

### 3.15. Consumer Survey

#### 3.15.1. Sociodemographic Profile of Respondents

**Figure 8:**
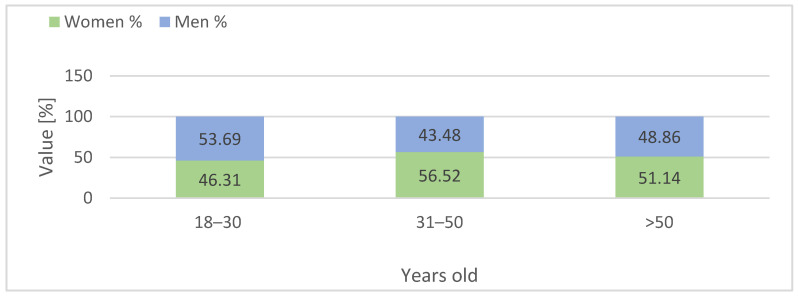
Percentage [%] of respondents consuming goose meat by survey response category. Source: own elaboration based on survey results.

**Figure 9:**
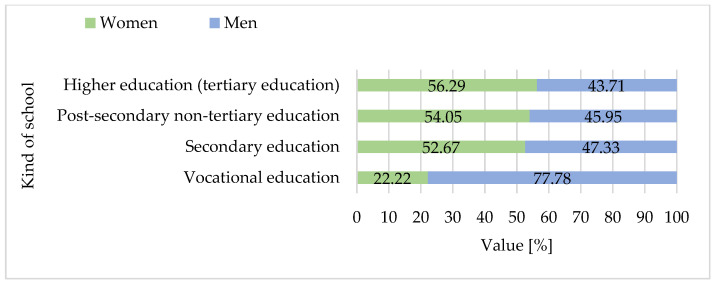
Percentage [%] of respondents consuming goose meat by survey response category. Source: own elaboration based on survey results.

**Figure 10:**
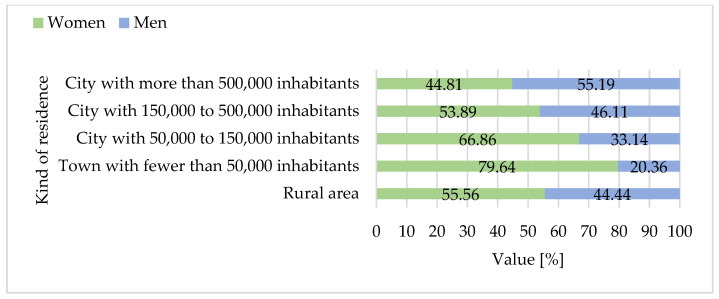
Percentage [%] of respondents consuming goose meat by survey response category. Source: own elaboration based on survey results.

The consumer survey conducted between 2022 and 2025 included a nationally diverse sample of 1.036 adult Polish residents aged 18 and above. The majority of respondents were under the age of 50, with nearly half falling into the 18–30 age group and a comparable share in the 31–50 bracket. Only a small proportion of participants were over 50 (Figure 8). Across all age groups, women were slightly more represented than men. Almost half of the respondents had completed secondary education, while a similarly large portion held higher (tertiary) degrees. Vocational or post-secondary non-tertiary qualifications were reported by only a small minority (Figure 9). In terms of place of residence, more than half of the participants lived in major urban centers with populations exceeding 500,000. Roughly one-third resided in medium or small cities, while rural residents comprised only a modest share of the sample (Figure 10).

The study included 1.036 participants, of whom 561 (54.1%) were women and 475 (45.9%) were men. Participants’ ages ranged from 18 to over 50 years, with a mean age of 34.4 years (SD = 11.1). The largest proportion of respondents was aged 18–30 (47.1%), followed by those aged 31–50 (44.4%), and over 50 (8.5%). The mean age was 33.3 years (SD = 10.8) for women and 34.2 years (SD = 11.3) for men (own calculations based on data from Figure 8).

The consumer survey reveals a persistent structural detachment between Poland’s strong export specialization in goose meat and its weak domestic consumption base. The overwhelming majority of respondents (66.6%) reported never having consumed goose meat in their lives, while only 1.4% declared regular consumption (at least once per month). Occasional consumption (a few times per year) was reported by 22.3% of participants, and a further 9.7% indicated having tried it once or twice. These findings confirm the depth of the domestic market gap, supporting hypothesis H3, which posited that cultural, informational, and economic factors act as significant consumption barriers despite Poland’s position as a global export leader in goose products. Consumption, where it does occur, is heavily ritualized and seasonal. Among those who consume goose meat, the majority associate it with St. Martin’s Day in November, suggesting a narrow temporal window for domestic demand, embedded in local tradition rather than integrated into daily or weekly dietary practices. Very few respondents cited occasions such as Christmas, Easter, or dining out as regular consumption contexts, and only 9% mentioned restaurant settings as a place for goose meat intake. This aligns with earlier studies by Szafrańska et al. (2021), who also noted that among surveyed respondents, goose meat is considered as a festive and symbolic dish, rather than a product of everyday relevance [36].

Barriers to broader adoption are multifaceted. Price remains a dominant factor: nearly one in four respondents (23.7%) indicated that they would be more likely to purchase goose meat if it were cheaper. However, economic accessibility is not the sole challenge. Many participants reported practical obstacles such as a lack of availability in stores, absence of ready-to-cook products, or insufficient culinary knowledge. Notably, more than half of all respondents (*n* = 521) stated explicitly that they were simply not interested in goose meat, regardless of price or accessibility, indicating a deeper cultural and psychological distance from the product.

The gender distribution of consumption patterns also reflects broader demographic and lifestyle dynamics. While men were slightly more represented among those purchasing meat in butcher shops or supermarkets, women more frequently cited factors such as lack of recipes or unfamiliarity with preparation methods as reasons for not purchasing. This gendered differentiation in perceived barriers resonates with the findings of [37], who found that women tend to be more cautious in adopting less familiar meat products and more responsive to health or culinary information.

#### 3.15.2. Consumption Behavior and Frequency of Eating Goose Meat in Poland

In terms of market channels, the survey revealed that goose meat remains largely absent from direct-to-consumer pathways. None of the respondents reported purchasing it online or at farmers’ markets, and the vast majority declared that they simply did not buy goose meat at all. Supermarkets were the most commonly mentioned source, but even there, purchases were rare and largely incidental. A particularly striking insight emerges from the responses related to consumer awareness and knowledge. Most respondents could not identify regional goose meat products, and few recalled seeing any promotional campaigns. This indicates that the existing promotional efforts have had a limited impact on public recognition or engagement. Similarly, findings from the study [38] revealed that a small percentage of respondents considered the country of origin as the primary factor when purchasing meat. This suggests that, although such information is available, it is not a key consideration for most surveyed consumers. Consequently, promotional strategies based solely on origin labeling may have limited effectiveness in attracting surveyed consumer attention or influencing purchasing decisions. Together, the survey results point to a profound disconnect between production and perception. Goose meat in Poland remains a product of export value but low domestic visibility, both in culinary practice and public consciousness. Overcoming this disconnect will require not only price adjustments or increased distribution but also a comprehensive reframing of goose meat as a healthy, sustainable, and culturally relevant choice for modern consumers.

**Figure 11:**
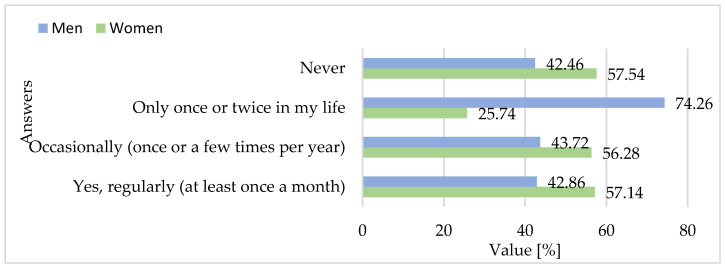
Have you ever eaten goose meat? Source: own elaboration based on survey results.

Fisher’s exact tests were used to compare categorical responses between women and men. Regarding past consumption of goose meat, no significant gender differences were found for regular consumption (*p* = 1.0) or occasional consumption (*p* = 0.50). However, men were significantly more likely than women to report having eaten goose meat only once or twice in their life (*p* < 0.001), while women were more likely to report never having consumed goose meat (*p* = 0.002) (Figure 11).

**Figure 12:**
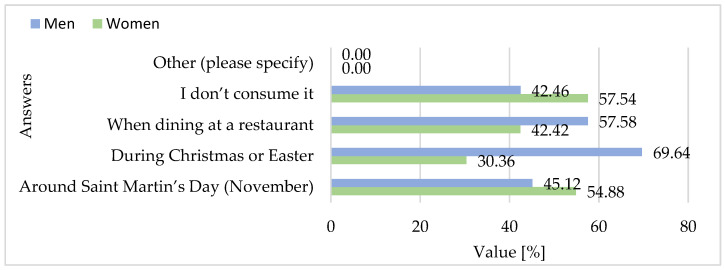
When do you usually consume goose meat? *. Source: own elaboration based on survey results. * Items marked with an asterisk were multiple-response questions, permitting respondents to select more than one option.

Moreover, it should be noted that a significant number of consumers who eat goose meat explicitly state that it is most often eaten during Christmas or Easter, when they are in a restaurant, or, according to tradition, around St. Martin’s Day (Figure 12).

**Figure 13:**
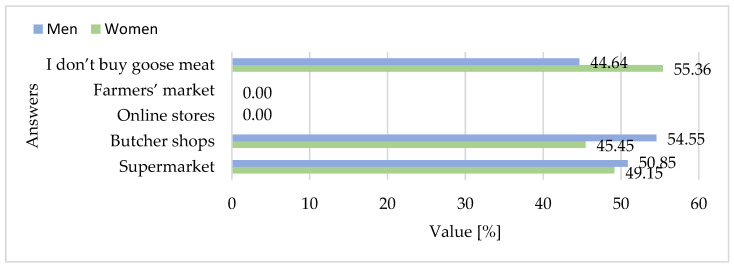
Where do you usually buy meat products (including goose meat)? *. Source: own elaboration based on survey results. * Items marked with an asterisk were multiple-response questions, permitting respondents to select more than one option.

When asked about purchasing habits (Figure 13), no statistically significant differences were observed between women and men for supermarket purchases (*p* = 0.087), butcher shop purchases (*p* = 0.52), or reporting that they do not buy goose meat (*p* = 0.19).

In terms of factors that could encourage increased consumption, women were significantly more likely than men to indicate that ready-to-cook products (*p* < 0.001) and more recipes or instructions for preparation (*p* < 0.001) would motivate them. Conversely, men were more likely to report no interest in increasing consumption (*p* < 0.001). No significant gender differences were found for other motivating factors, including lower price, greater availability in stores, or more information about health benefits.These results suggest that gender influences specific attitudes and practical barriers to goose meat consumption, particularly in relation to convenience and engagement with preparation methods, whereas general purchasing behavior shows less pronounced gender differences.

**Figure 14:**
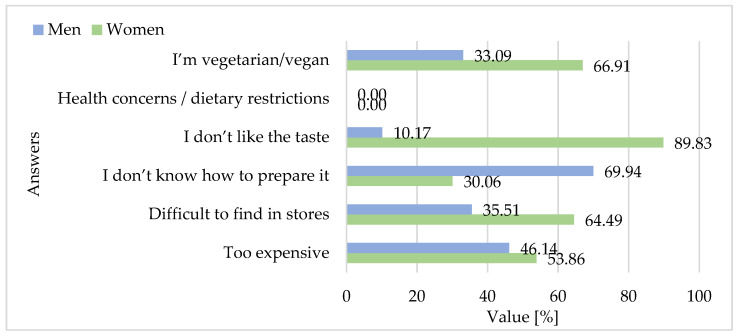
What are the main reasons why you don’t consume goose meat more often? *. Source: own elaboration based on survey results. * Items marked with an asterisk were multiple-response questions, permitting respondents to select more than one option.

**Figure 15:**
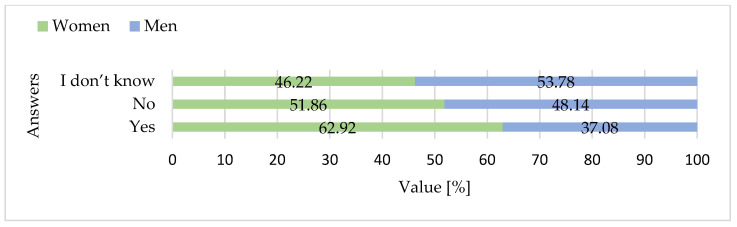
In your opinion, is goose meat healthier than other types of poultry (e.g., chicken, duck, turkey)? Source: own elaboration based on survey results.

**Figure 16:**
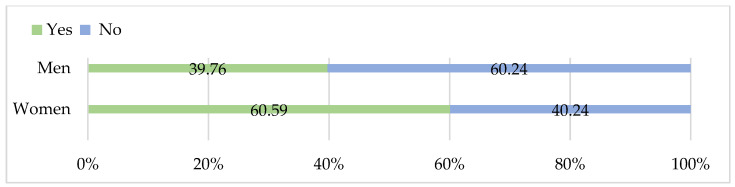
Have you noticed any promotional campaigns related to Polish goose meat in recent years? Source: own elaboration based on survey results.

**Figure 17:**
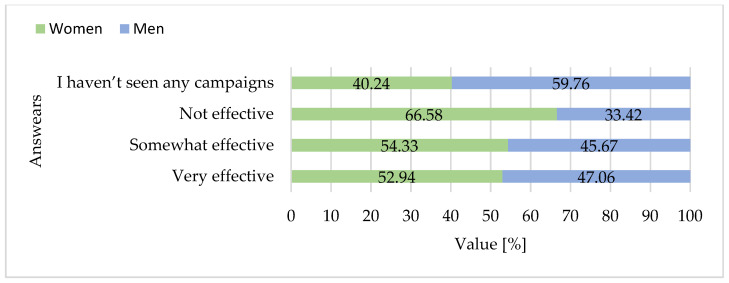
How effective do you think these campaigns are in promoting domestic goose meat consumption? Source: own elaboration based on survey results.

Figure 14, Figure 15, Figure 16 and Figure 17 provide valuable insights into the structural challenges shaping the domestic goose meat market in Poland. The survey results confirm that among surveyed consumers face a combination of economic, logistical, and informational barriers to consumption. The most frequently cited obstacle remains the high price, which positions goose meat as a luxury item rather than a staple protein source. Equally critical is the limited availability of products in retail chains, which reflects both a supply constraint and the overwhelming export orientation of the sector. 

On the nutritional side, awareness is fragmented. Only about one-third of respondents recognized the favorable health attributes of goose meat, while another third denied or were unsure about its potential benefits (Figure 14). 

Since more than 90% of national production is directed to foreign markets, predominantly Germany and other Western European countries, the domestic market is structurally undersupplied, reinforcing consumer perceptions of goose meat as rare and inaccessible. This scarcity is further compounded by culinary unfamiliarity—around one-quarter of respondents openly admitted not knowing how to prepare goose meat (Figure 14). The lack of recipes, tutorials, or convenient product formats creates an additional barrier to market penetration.

The knowledge gap suggests that the proven dietary advantages of goose meat, such as its high share of unsaturated fatty acids, are not reaching the general public in an accessible and credible way. Consumers do not realize that goose meat is healthier in terms of nutritional value than other types of poultry (Figure 15). 

For the food technology sector, these findings carry clear implications. The structural imbalance between high export volumes and limited domestic supply can only be corrected through a dual strategy. On the one hand, technological innovation is needed to lower consumer barriers, for example, by developing portioned, marinated, or ready-to-cook goose products that appeal to less experienced cooks. On the other hand, nutrition education and communication must be intensified to strengthen consumer awareness of goose meat’s unique health profile. At the same time, supply chain adjustments could increase domestic availability without undermining export competitiveness, for instance by valorizing by-products or diversifying into processed categories (pâtés, sausages, ready meals) aimed at local consumers.

In this sense, Figure 14, Figure 15, Figure 16 and Figure 17 not only illustrate the persistence of cultural and informational barriers but also reveal how the export-driven trade model indirectly exacerbates domestic demand constraints. By reallocating a fraction of production to the local market and aligning it with consumer-friendly formats, Poland could reduce its dependence on Western buyers while gradually fostering a more resilient and diversified food system.

Analysis of categorical survey responses using Fisher’s exact tests revealed several significant differences between women and men. The majority of participants reported noticing promotional campaigns for goose meat, with a higher proportion of women (60.6%) than men (39.4%) acknowledging such campaigns (*p* = 0.002) (Figure 16). Gender differences were particularly pronounced for reasons for not consuming goose meat. Women were significantly more likely than men to indicate that they found goose meat too expensive (53.9% vs. 46.1%, *p* = 0.045) and difficult to find in stores (64.5% vs. 35.5%, *p* < 0.001). Conversely, men were more likely to report not knowing how to prepare goose meat (69.9% vs. 30.1%, *p* < 0.001). A strong gender disparity was observed for the dislike of taste, with 89.8% of respondents indicating this barrier being women compared to only 10.2% men (*p* < 0.001) (Figure 14).

The prevalence of vegetarian or vegan respondents was higher among women (66.9%) than men (33.1%), and this difference was also statistically significant (*p* = 0.012). No significant gender differences were found for perceptions of the environmental sustainability of goose meat (*p* = 0.785) (Figure 14).

These findings highlight that gender plays a significant role in shaping both practical and attitudinal barriers to goose meat consumption, with women generally reporting higher awareness of promotional campaigns and more frequently citing price and availability as limiting factors, while men reported a lack of knowledge regarding preparation as the primary barrier.

## 4. Discussion

### 4.1. Export Concentration and Systemic Vulnerability (H2)

Hypothesis H2 posited that Poland’s goose exports are geographically concentrated to a degree that compromises sectoral resilience. The data strongly support this proposition. Germany consistently absorbed between 74.2% and 85.8% of Poland’s total goose meat export value throughout 2020–2024, with the highest concentration occurring in 2022. The corresponding Herfindahl–Hirschman Index exceeds 5500 points, far surpassing the 2500-point threshold typically considered indicative of highly concentrated markets. This concentration substantially exceeds levels typically observed in agricultural commodity trade and creates significant exposure to demand shocks, policy changes, and market disruptions [39,40,41].

The economics literature on supply chain resilience emphasizes that diversification across both products and destinations serves as a primary risk mitigation strategy. Wood et al. [42] argue that food system resilience requires balanced integration across local, regional, and global scales rather than overwhelming dependence on any single market level. The Polish goose sector’s near-total orientation toward external markets contradicts principles of food system resilience that emphasize the importance of domestic consumption capacity as a buffer against international market volatility. Recent analysis of food system resilience measurement frameworks highlights that systems demonstrating strong export specialization without commensurate domestic market development exhibit reduced capacity to absorb external shocks [43]. Our empirical findings map onto this framework—the relative stability of export volumes despite the 2022 price shock suggests absorptive capacity, while the limited diversification of export destinations and the underdeveloped domestic market indicate constrained adaptive capacity.

A critical finding is that concentration increased during the period of peak prices (2021–2022), rising from 74.2% to 85.8%. This pattern indicates that price-driven demand shifts did not trigger geographic diversification; rather, the German market absorbed an even larger share of Polish exports during the crisis period. The implication is troubling: under conditions of market stress, the sector’s dependence on its dominant buyer intensified rather than diminished. This concentration risk is particularly acute given evolving geopolitical and economic uncertainties affecting European trade. Recent disruptions to agricultural supply chains, including those stemming from climate events, pandemic-related logistics constraints, and geopolitical tensions, underscore the vulnerability of highly concentrated export models [44]. This finding is robust to alternative aggregation choices. In 2022, when Germany’s share peaked at 85.8%, the squared share of Germany alone (0.858 ≈ 7362) accounts for the vast majority of the index value; the second-largest partner (France, at 4.3%) contributes only approximately 18 additional points. Excluding or aggregating all minor destinations (individually below 1% of export value) does not materially alter the HHI, which remains above 5500 in every year of the study period, regardless of how the smallest trade partners are treated.

Comparative positioning within broader agricultural trade patterns illuminates both unique characteristics and common challenges. While Hungary exhibits comparable export values (EUR 78 million in 2023, EUR 62 million in 2024), no other EU member state exceeded EUR 5 million annually. Globally, only Poland, China, and Hungary reported export values exceeding USD 80 million in 2023, confirming Poland’s exceptional position in this specialized market. However, this leadership position is built upon structural dependencies that warrant strategic attention.

### 4.2. Price Dynamics and Limited Demand Responsiveness (H1)

Hypothesis H1 anticipated that export volumes would prove limited price responsiveness with respect to exchange-rate movements and price fluctuations. The observed patterns, specifically volume stability despite near-tripling of unit prices, confirm this expectation, though interpretation requires careful attention to simultaneous supply-side shocks. Export prices in euros nearly doubled in 2022 compared to 2020 (an increase of approximately 195%), while export volumes declined only modestly from 19.5 to 14.6 million kg. By 2024, prices had returned to levels observed in 2020–2021, yet volumes recovered to 15.0 million kg. This pattern is consistent with low-demand limited price responsiveness in the German market.

Standard agricultural economics suggests that most agricultural products exhibit limited price responsiveness due to the availability of substitutes [45]. However, specialty products with unique characteristics can achieve differentiated market positions that reduce price sensitivity. The maintenance of export volumes during the 2022 price surge indicates that Polish goose meat occupies a specialized niche in German consumption patterns that cannot be easily substituted. This finding aligns with economic theory on quality-differentiated agricultural trade, where production methods, geographic origin, and specific breed characteristics create product attributes that competitors cannot readily replicate.

The White Koluda breed’s specific characteristics, including the traditional oat-based fattening system, create sensory and nutritional attributes valued by German consumers. This product differentiation provides pricing power but simultaneously constrains market expansion potential, as these attributes appeal primarily to consumers already familiar with traditional goose consumption practices. Product-level analysis reveals heterogeneous price movements: frozen whole carcasses (CN 0207 52 90) and frozen breasts with bone (CN 0207 55 51) exhibited the largest price increases (over 250% and 346% of base-year values, respectively) while maintaining substantial volume. This suggests that premium product categories demonstrate particularly robust demand.

Exchange-rate insensitivity provides additional confirmation of the differentiation effect. The EUR/PLN exchange rate rose in 2022 by merely 5% compared to 2020, followed by a decrease of approximately 8% by 2024, movements insufficient to explain the observed price dynamics. Standard trade theory predicts that currency depreciation should stimulate export volumes through improved price competitiveness. The absence of such a response indicates that quality attributes rather than price competitiveness drive demand. This phenomenon has also been observed in other specialty agricultural products, where terroir, production methods, or breed-specific characteristics lead to limited price responsiveness among targeted consumer segments.

Caution is warranted in interpreting these patterns as formal, limited price responsiveness estimates. The 2022 price surge was driven primarily by supply-side factors—specifically avian influenza reducing flock sizes across Europe (predominantly affecting France with 68% of outbreaks and Hungary with 24%), combined with feed cost increases exceeding 33%—rather than demand shifts. In such circumstances, observed price-quantity combinations do not trace out a demand curve from which limited price responsiveness could be estimated; they reflect supply curve shifts.

### 4.3. The Domestic Consumption Gap: Survey Evidence (H3)

Hypothesis H3 anticipated persistent domestic under-consumption despite global export leadership. Survey evidence confirms this gap and identifies its multidimensional drivers. The overwhelming majority of respondents (66.6%) reported never having consumed goose meat, while only 1.4% declared regular consumption (at least once per month). Occasional consumption (a few times per year) was reported by 22.3% of participants. These findings confirm the depth of the domestic market gap and demonstrate that cultural, informational, and economic factors act as significant consumption barriers despite Poland’s position as a global export leader.

Consumption, where it does occur, is heavily ritualized and seasonal. Among those who consume goose meat, the majority associate it with St. Martin’s Day in November, suggesting a narrow temporal window for domestic demand embedded in local tradition rather than integrated into daily dietary practices. Very few respondents cited Christmas, Easter, or dining out as regular consumption contexts. This pattern parallels findings in research on traditional food products, where strong cultural associations can simultaneously sustain and constrain markets by reinforcing perception of products as occasional rather than routine purchases [46,47].

Barriers to broader adoption are multifaceted. Price remains a dominant factor: nearly one in four respondents (23.7%) indicated that lower prices would increase purchase likelihood. However, economic accessibility is not the sole challenge. Many participants reported practical obstacles such as limited availability in stores, absence of ready-to-cook products, and insufficient culinary knowledge. Women were significantly more likely than men to indicate that ready-to-cook products (*p* < 0.001) and more recipes or instructions (*p* < 0.001) would motivate increased consumption. Notably, 43.7% of respondents stated explicitly that they were simply not interested in goose meat regardless of price or accessibility, indicating a deeper cultural and psychological distance from the product that cannot be addressed through pricing or distribution alone.

Awareness of goose meat’s nutritional attributes is fragmented. Only about one-third of respondents recognized the favorable health characteristics, while another third denied or were uncertain about potential benefits. The documented favorable fatty acid profile of oat-fed goose meat provides defensible health positioning, yet this attribute remains largely unknown to potential consumers. Research on European meat markets during 2023 identified health considerations as dominant factors influencing purchasing decisions [48], suggesting significant untapped positioning potential if nutrition communication can be strengthened.

Market channel analysis reveals a structural absence from direct-to-consumer pathways. None of the respondents reported purchasing goose meat online or at farmers’ markets, and the majority declared that they simply did not buy goose meat at all. Supermarkets were the most commonly mentioned source, but even there, purchases were rare and incidental. Most respondents could not identify regional goose meat products and few recalled seeing promotional campaigns, indicating that existing promotional efforts have had a limited impact on public recognition or engagement.

### 4.4. Observed Interdependencies: Linking Concentration, Prices, and Consumption

The three empirical domains examined above—export concentration, price dynamics, and domestic consumption—display patterns that appear mutually reinforcing rather than independent. The data do not permit formal causal identification of these linkages; however, the co-occurrence of high geographic concentration, limited price responsiveness, and negligible domestic demand forms a consistent pattern that warrants interpretive synthesis. This section draws together the findings to articulate these observed interdependencies as a plausible analytical framework.

#### 4.4.1. High Geographic Concentration and Indicators of Demand-Shock Vulnerability

With Germany absorbing 74–86% of exports, Polish producers operate within a highly concentrated buyer structure consistent with monopsonistic conditions. When German demand contracted in 2023–2024, total export value fell 47% from its 2022 peak (EUR 118.0 million to EUR 62.7 million), a decline absorbed entirely by Polish producers with no alternative market of comparable scale. The documented limited price responsiveness (Section 4.2) suggests that volume adjustments, rather than price adjustments, may bear the brunt of demand fluctuations. If this interpretation holds, the resulting asymmetry would effectively transfer market risk from buyers to producers—a hypothesis that merits formal testing with higher-frequency data.

#### 4.4.2. The Absence of Domestic Demand Removes a Natural Absorption Buffer for Production Fluctuations

In agricultural sectors with developed home markets, domestic consumption can absorb surplus production during periods of weak export demand, stabilizing producer revenues and preventing distress sales. With Polish per capita consumption below 0.3 kg annually, compared to approximately 95% of production directed to export, this buffering mechanism is effectively absent. Survey evidence (Section 4.3) demonstrates that this gap is not merely a function of insufficient supply to domestic channels; rather, deep-seated cultural unfamiliarity and informational barriers suppress latent demand. The sector thus operates without a safety valve that could moderate the impact of external market volatility.

#### 4.4.3. Consumer Barriers Perpetuate Export Dependence Through a Self-Reinforcing Cycle

Because domestic demand remains undeveloped, producers have little incentive to invest in consumer-oriented product innovation, retail distribution, or marketing. This underinvestment, in turn, reinforces consumer unfamiliarity and maintains the barriers documented in Section 4.3. The result is path dependence, because the export-oriented model, once established, becomes increasingly difficult to diversify because the capabilities and relationships required for domestic market development atrophy through disuse.

This structural configuration embodies both the strengths and vulnerabilities of a highly specialized agri-food industry operating in a globalized environment. The sector achieves strong economic performance through export specialization and quality differentiation, but demonstrates limited social integration through domestic consumption and faces potential environmental challenges through cold chain energy requirements and transport distances. The dominant reliance on frozen products for export generates substantial energy requirements throughout cold chain distribution, while the geographic displacement of consumption from production contradicts principles of food system localization that have gained prominence in sustainability discourse [49,50,51,52]. However, this perspective must be balanced against economic realities: Polish goose producers face limited domestic demand and rely on export markets for economic viability.

### 4.5. Policy Implications and Strategic Directions

The patterns we observe are consistent with the following policy considerations.

#### 4.5.1. Reducing Concentration Risk Through Export-Market Diversification

The HHI exceeding 5500 and the documented vulnerability when German demand contracted (47% value decline from peak) establish the empirical case for diversification. The intermittent presence of Hong Kong (fluctuating from 8.2% in 2020 to 0.8% in 2022 before recovering to 5.7% in 2024) and the emergence of Canada (0.7% in 2024) indicate initiated relationships that could be systematically developed. Asian markets, particularly in countries with established goose consumption traditions, represent logical expansion targets [53]. China accounts for approximately 95% of global goose production but maintains different production systems; this suggests potential for Polish products positioned as premium alternatives for high-income Asian consumer segments. France’s share increased from 4.3% to 7.1% over the study period, demonstrating that even within Europe, diversification opportunities exist. However, market development in distant regions requires sustained investment in distribution infrastructure, regulatory compliance, and brand building that may exceed individual producer capacity, warranting coordinated industry and governmental support.

#### 4.5.2. Stimulating Domestic Demand Through Product Innovation and Nutrition Communication

Survey findings identify specific, addressable barriers. The 23.7% of respondents citing price sensitivity and the significantly higher proportion of women indicating that ready-to-cook products and recipes would motivate consumption (*p* < 0.001 for both) point toward product innovation as a viable strategy. Developing portioned, marinated, or ready-to-cook goose products could lower adoption barriers while commanding premium pricing. Recent trends in European poultry markets show strong consumer interest in convenient, ready-to-eat products, with new product launches in these categories showing double-digit growth [54]. The fragmented awareness of nutritional attributes (only one-third recognizing health benefits) indicates substantial scope for nutrition communication. The favorable fatty acid profile documented in multiple studies provides defensible health positioning; communicating this advantage credibly could shift consumer perceptions. Valorizing by-products through processed categories (pates, sausages, ready meals) aimed at local consumers could increase domestic availability without undermining export competitiveness.

#### 4.5.3. Enhancing Value Capture Through Quality Signaling and Branding

The limited price responsiveness documented in Section 4.2 confirms that German consumers value the distinctive attributes of Polish goose meat. This finding supports investment in geographical-indication labeling, premium positioning, and brand building to formalize and protect the quality differentiation that currently operates informally. The White Koluda breed’s inclusion in the List of Traditional Products by the Ministry of Agriculture (August 2023) provides a foundation for such positioning. Existing promotional campaigns (Gans und Ente aus Polen in Germany since 2021; Kachna a Husa z Polska in the Czech Republic since 2024) represent initial steps, but survey evidence suggests limited domestic awareness of these efforts. Strengthening the integration between export-oriented branding and domestic market development could create synergies currently unexploited.

### 4.6. Limitations and Future Research

Several limitations warrant acknowledgment. Regarding data structure, annual aggregation masks within-year seasonality and monthly variation that may affect the interpretation of price-volume relationships. Goose meat consumption concentrates heavily in the November-December period, and annual data cannot capture these dynamics. Some CN product categories, particularly residual groupings like CN 0207 55 81 and CN 0207 55 99, may contain heterogeneous products; interpretations of these categories should be appropriately cautious. Minor discrepancies (typically less than 2%) exist between GUS, Eurostat, and UN Comtrade data due to timing differences in reporting and CIF/FOB valuation conventions; we cross-validated sources where overlapping.

Regarding survey methodology, the non-probability CAWI sampling and urban overrepresentation (over half of respondents from cities exceeding 500,000 population) limit generalizability. Findings should be interpreted as indicative of patterns among the sampled population. Self-selection bias may further skew results. While model-based inference with robust standard errors was applied where appropriate, the exploratory nature of the survey component should be recognized.

Most fundamentally, the descriptive approach cannot establish causal relationships. The observed association between price stability and volume maintenance is consistent with low demand limited price responsiveness but could reflect multiple confounded factors, including simultaneous supply constraints, contractual arrangements, or measurement artifacts. The structural interdependencies articulated in Section 4.4 represent a plausible interpretive framework rather than causally identified relationships.

Several research avenues merit future investigation. Comparative analysis with other European goose producers, particularly Hungary and France, would illuminate whether concentration patterns reflect sector-wide dynamics or Poland-specific factors. Hungary’s position as both a major producer and primary supplier to Poland’s import market creates interesting competitive and complementary dynamics, warranting detailed examination. Consumer segmentation analysis could identify niche markets more receptive to goose meat adoption; rather than treating domestic consumers as homogeneous, targeted research identifying psychographic or demographic segments with higher affinity could enable more efficient market development. Value chain analysis incorporating producer, processor, distributor, and retailer perspectives would provide a more complete understanding of where economic value accumulates and where intervention points exist for improving producer returns or consumer access [55,56]. There are several strategic directions for increasing the resilience and sustainability of the food system while maintaining export competitiveness. First, developing the domestic market represents an untapped opportunity to mitigate risk. The study’s findings indicate that barriers combine economic, informational, and cultural dimensions, requiring coordinated intervention rather than single-factor solutions. Product innovation toward convenience formats, pre-marinated cuts, ready-to-cook portions, and value-added preparations could lower adoption barriers while commanding premium pricing. Recent trends in European poultry markets show strong consumer interest in convenient, ready-to-eat products (for example, sous-vide) [54], with new product launches in these categories showing double-digit growth. Nutritional communication strategies merit strengthening. The favorable fatty acid profile of oat-fed goose provides defensible health positioning, yet this attribute remains largely unknown. Recent consumer research indicates that health considerations rank among the top of meat purchasing decisions across European markets, suggesting significant positioning potential [56].

## 5. Conclusions

This study provides the first comprehensive, multi-year economic assessment (2020–2024) of Poland’s foreign trade in goose meat and edible offal, revealing a sector characterized by exceptional international competitiveness but persistent domestic marginalization. Poland remains one of the world’s top exporters, alongside China and Hungary, thanks to its specialization in the White Kołuda^®^ breed, efficient production systems, and well-established commercial relations, particularly with Germany. Exports peaked at €118 million in 2022; the observed maintenance of substantial export volumes despite near-tripling of unit prices is consistent with limited price responsiveness in the German market, though formal elasticity estimation is precluded by simultaneous supply-side disruptions. However, the sector’s structural dependence on a single foreign market (Germany ≈ 75–85% of exports) and on a narrow product range (frozen cuts ≈ 60–65% of value) exposes producers to significant systemic risk. Tied to HHI calculation and documented vulnerability when German demand contracted in 2023–2024 (export value fell 47% from peak).

Domestic consumption, by contrast, remains extremely low (<0.3 kg per capita annually) and culturally peripheral. Survey evidence confirms that two-thirds of surveyed consumers have never eaten goose meat and that awareness of its nutritional or culinary value is limited. These findings underscore a deep disconnection between a globally competitive export industry and an underdeveloped domestic market. Price incentives alone cannot overcome cultural and informational barriers; sustained consumer education, retail availability, and product innovation are required to normalize goose meat as an everyday food rather than a seasonal delicacy, as evidenced by survey findings showing that 66.6% of respondents had never consumed goose meat and other specific barriers (price, availability, culinary unfamiliarity).

From a policy and strategic perspective, the data highlight the need to balance export specialization with diversification and domestic integration. Expanding beyond the German market, particularly toward Asian destinations with established culinary demand, could strengthen food system resilience, though such expansion requires coordinated industry and governmental support. Concurrently, stimulating local demand through marketing campaigns, convenience-oriented products, and gastronomic education would enhance internal market stability and align with broader sustainability goals.

Poland’s goose meat sector embodies both the strengths and vulnerabilities of a highly specialized agri-food industry operating in a globalized environment. Its future competitiveness will depend on maintaining technological and quality advantages while building resilience through market diversification, value-added processing, and domestic demand development. Achieving this balance offers the most promising path toward a robust, adaptive, and sustainable Polish goose industry.

## Figures and Tables

**Table 1:** Polish exports of meat and edible offal of geese in the years 2020–2024, by country share (in %).

Country	2020	2021	2022	2023	2024
Germany	74.2	84.0	85.8	78.1	74.4
France	5.3	4.8	4.3	6.0	7.1
Hong Kong	8.2	4.0	0.8	4.0	5.7
Czechia	5.4	2.7	1.0	2.8	3.4
UK	1.5	1.2	1.0	1.4	2.5
Austria	1.4	0.9	1.7	2.4	2.4
Italy	0.2	0.4	0.8	2.6	0.9
Denmark	0.7	0.3	0.9	0.7	0.8
Canada	0.0	0.0	0.0	0.0	0.7
Romania	0.7	0.2	0.4	0.3	0.3
China	0.0	0.2	0.1	0.0	0.3
Hungary	1.0	0.1	1.9	0.3	0.2
Sweden	0.1	0.1	0.0	0.1	0.2
Slovenia	0.0	0.0	0.0	0.1	0.2
Singapore	0.0	0.0	0.0	0.0	0.2
Netherlands	0.2	0.3	0.5	0.3	0.1
Slovakia	0.3	0.1	0.3	0.2	0.1
Ireland	0.0	0.1	0.1	0.2	0.1
Estonia	0.2	0.1	0.1	0.1	0.1
Belgium	0.3	0.1	0.1	0.0	0.1
Lithuania	0.1	0.2	0.0	0.0	0.0
Others	0.2	0.2	0.2	0.4	0.2

Source: own development based on [23].

**Table 2:** Polish exports of meat and edible offal of geese in the years 2020–2024, by product group and by country (in mln EUR).

Product Group	Country	2020	2021	2022	2023	2024
Meat, not cut into pieces, fresh or chilled	Germany	0.8	1.0	1.3	0.8	0.5
	Czechia	0.7	0.5	0.0	0.3	0.2
	UK	0.0	0.0	0.1	0.3	0.0
	Denmark	0.1	0.2	0.2	0.1	0.0
	Romania	0.1	0.0	0.2	0.0	0.0
	Others	0.2	0.1	0.1	0.0	0.2
Meat, not cut into pieces, frozen	Germany	10.1	22.7	34.1	18.9	12.2
	UK	0.5	0.4	0.8	0.9	1.5
	France	0.0	0.2	0.6	1.5	1.3
	Austria	0.5	0.3	1.1	0.8	0.8
	Czechia	0.8	0.9	0.7	0.8	0.7
	Denmark	0.1	0.0	0.8	0.5	0.4
	Italy	0.0	0.1	0.2	0.3	0.2
	Hungary	0.2	0.0	1.2	0.2	0.1
	Ireland	0.0	0.1	0.2	0.1	0.1
	Slovenia	0.0	0.0	0.0	0.1	0.1
	Sweden	0.0	0.0	0.0	0.1	0.1
	Canada	0.0	0.0	0.0	0.0	0.1
	Netherlands	0.0	0.1	0.2	0.0	0.0
	Belgium	0.0	0.0	0.1	0.0	0.0
	Others	0.3	0.2	0.3	0.2	0.2
Meat, in cuts, fresh or chilled	Germany	2.6	2.2	3.6	2.5	1.6
	Czechia	0.4	0.4	0.0	0.3	0.3
	Hong Kong	0.5	0.2	0.1	0.2	0.2
	Italy	0.0	0.0	0.3	0.2	0.1
	France	0.1	0.0	0.0	0.0	0.0
	Others	0.2	0.1	0.1	0.0	0.2
Meat, in cuts, frozen	Germany	25.7	37.1	61.7	46.0	31.9
	France	2.4	3.1	4.2	3.5	3.1
	Hong Kong	2.8	2.1	0.5	2.3	2.8
	Czechia	0.7	0.2	0.3	0.8	0.7
	Austria	0.2	0.3	0.9	1.2	0.6
	Canada	0.0	0.0	0.0	0.0	0.3
	Italy	0.1	0.2	0.5	1.8	0.2
	China	0.0	0.1	0.1	0.0	0.2
	UK	0.3	0.5	0.2	0.1	0.1
	Netherlands	0.1	0.1	0.3	0.2	0.0
	Hungary	0.3	0.0	0.9	0.1	0.0
	Slovakia	0.0	0.0	0.1	0.1	0.0
	Spain	0.0	0.1	0.1	0.0	0.0
	Belgium	0.1	0.0	0.1	0.0	0.0
	Romania	0.1	0.0	0.0	0.0	0.0
	Others	0.1	0.1	0.0	0.2	0.2
Edible offal, frozen	Hong Kong	1.1	0.8	0.4	1.0	0.5
	Germany	0.4	0.5	0.7	0.6	0.4
	Czechia	0.2	0.1	0.2	0.2	0.2
	France	0.3	0.3	0.2	0.3	0.1
	Romania	0.1	0.2	0.1	0.2	0.1
	Danmark	0.1	0.0	0.1	0.0	0.0
	Others	0.2	0.1	0.1	0.2	0.2
Total		53.5	75.6	118.0	87.9	62.7

Source: own development based on [23].

**Table 3:** Polish imports of meat and edible offal of geese in the years 2020–2024, by product group and by country of origin (in thousand EUR).

Product Group	Country	2020	2021	2022	2023	2024
Meat, not cut into pieces, fresh or chilled	Slovakia	22.6	0.0	0.0	0.0	0.0
	Others	0.0	0.0	0.0	0.0	0.0
Meat, not cut into pieces, frozen	Hungary	0.0	0.0	0.0	120.2	0.0
	Germany	0.7	0.0	0.0	0.0	0.0
Meat, in cuts, fresh or chilled	Hungary	0.0	0.0	0.1	0.0	29.4
	Germany	5.2	0.0	0.0	46.1	0.0
	Netherlands	0.0	0.0	0.0	3.4	0.0
	France	4.9	0.0	0.0	0.0	0.0
Meat, in cuts, frozen	Hungary	0.0	41.0	174.9	105.7	191.4
	Germany	4.3	55.5	87.3	20.6	0.0
Edible offal, fresh or chilled	Hungary	0.0	0.0	0.0	0.1	0.0
	France	0.7	0.0	0.0	0.0	0.0
Edible offal, frozen	France	0.8	8.4	1.1	1.9	4.0
	Spain	0.1	0.1	1.8	2.1	0.7
	Hungary	7.5	67.1	205.1	137.5	0.0
	Slovakia	0.0	0.0	1.3	0.0	0.0
	Germany	0.0	0.8	0.2	0.0	0.0
Total	Total	46.9	172.9	471.9	437.4	225.6

Source: own development based on [23].

**Table 4:** Polish exports of meat and edible offal of geese in the years 2020–2024, by major CN product group.

CN Code	Description	Value (in Million EUR)					Volume (in Thousand Tons)				
		2020	2021	2022	2023	2024	2020	2021	2022	2023	2024
0207 51 90	Meat, not cut into pieces, fresh or chilled: plucked and drawn, without heads and feet (“75% geese”)	1.9	1.8	1.9	1.5	0.9	0.6	0.4	0.2	0.2	0.2
0207 52 90	Meat, not cut into pieces, frozen: plucked and drawn, without heads and feet (“75% geese”)	12.5	25.0	40.2	24.3	17.8	6.8	6.8	6.1	5.4	6.1
0207 54 41	Meat, in cuts, fresh or chilled, with bone: backs, necks, rumps, wing-tips	0.1	0.0	0.1	0.0	0.0	0.3	0.0	0.0	0.0	0.0
0207 54 51	Meat, in cuts, fresh or chilled, with bone: breasts	1.3	1.1	1.9	1.1	0.8	0.2	0.1	0.1	0.1	0.1
0207 54 61	Meat, in cuts, fresh or chilled, with bone: legs	1.7	1.5	1.7	1.7	1.2	0.3	0.2	0.2	0.2	0.2
0207 55 10	Meat, in cuts, frozen, boneless	3.9	5.2	5.4	5.1	4.9	1.0	1.1	0.7	0.6	0.9
0207 55 31	Meat, in cuts, frozen, with bone: whole wings	1.1	0.7	0.7	1.2	1.2	0.9	0.7	0.7	0.9	0.7
0207 55 41	Meat, in cuts, frozen, with bone: backs, necks, rumps or wing-tips	1.3	1.6	2.1	1.5	1.1	2.0	1.1	0.9	0.7	0.9
0207 55 51	Meat, in cuts, frozen, with bone: breasts	8.7	14.5	27.5	18.6	12.8	2.2	2.2	2.1	1.9	2.2
0207 55 61	Meat, in cuts, frozen, with bone: legs	17.0	20.6	33.3	28.8	18.7	3.7	2.9	2.6	3.3	2.8
0207 55 81	Meat, in cuts, frozen, with bone: other than halves, quarters, whole wings, backs, necks, rumps, wing-tips, breasts, legs or paletots	0.6	0.8	0.5	0.9	1.2	0.2	0.6	0.2	0.5	0.4
0207 55 95	Edible offal, frozen: non-fatty livers	0.6	0.5	0.7	0.6	0.3	0.3	0.1	0.1	0.1	0.1
0207 55 99	Edible offal, frozen: other than livers	1.5	1.2	0.8	1.5	1.1	0.7	0.6	0.4	0.6	0.4
	Others	1.3	1.1	1.2	1.1	0.7	0.3	0.5	0.3	0.2	0.0
	Total	53.5	75.6	118.0	87.9	62.7	19.5	17.3	14.6	14.7	15.0

Source: own development based on [23].

**Table 5:** Polish imports of meat and edible offal of geese in the years 2020–2024, by major CN product group.

CN Code	Description	Value (in Thou. EUR)					Volume (in Tons)				
		2020	2021	2022	2023	2024	2020	2021	2022	2023	2024
0207 51 90	Meat, not cut into pieces, fresh or chilled: plucked and drawn, without heads and feet (“75% geese”)	22.6	0.0	0.0	0.0	0.0	5.7	0.0	0.0	0.0	0.0
0207 52 90	Meat, not cut into pieces, frozen: plucked and drawn, without heads and feet (“75% geese”)	0.7	0.0	0.0	120.2	0.0	0.4	0.0	0.0	17.7	0.0
0207 54 21	Meat in cuts, fresh or chilled, with bone: halves or quarters	5.2	0.0	0.0	0.0	0.0	21.1	0.0	0.0	0.0	0.0
0207 54 31	Meat in cuts, fresh or chilled, with bone: whole wings	0.0	0.0	0.0	46.1	0.0	0.0	0.0	0.0	13.1	0.0
0207 54 81	Meat in cuts, fresh or chilled, with bone: other than halves, quarters, whole wings, backs, necks, rumps, wing-tips, breasts, legs or paletots	0.0	0.0	0.0	0.0	29.4	0.0	0.0	0.0	0.0	20.9
0207 55 10	Meat in cuts, frozen, boneless	0.0	0.0	87.3	0.0	48.4	0.0	0.0	14.5	0.0	17.7
0207 55 31	Meat in cuts, frozen, with bone: whole wings	0.0	35.1	0.0	0.0	102.3	0.0	21.8	0.0	0.0	124.4
0207 55 51	Meat in cuts, frozen, with bone: breasts	0.8	0.0	65.6	20.6	0.0	0.1	0.0	5.8	2.3	0.0
0207 55 61	Meat in cuts, frozen, with bone: legs	3.5	20.3	51.2	0.0	40.8	0.6	4.0	3.8	0.0	13.5
0207 55 81	Meat in cuts, frozen, with bone: other than halves, quarters, whole wings, backs, necks, rumps, wing-tips, breasts, legs or paletots	0.0	41.0	58.1	105.7	0.0	0.0	32.3	14.9	35.1	0.0
0207 55 99	Edible offal, frozen: other than livers	7.3	74.3	206.2	137.5	0.0	6.6	56.6	82.0	79.6	0.0
	Others	6.8	2.2	3.5	7.3	4.7	1.1	0.1	0.0	3.1	0.0
	Total	46.9	172.9	471.9	437.4	225.6	35.6	114.8	121.0	150.9	176.5

Source: own development based on [23].

**Table 6:** Average prices per kilogram in Polish foreign trade in meat and edible offal of geese in the years 2020–2024 (2020 = 100), by major CN product group.

CN Code	Description	Prices in EUR					Prices in PLN				
		2020	2021	2022	2023	2024	2020	2021	2022	2023	2024
Total	Exports	100.0	160.0	295.3	217.9	152.7	100.0	164.2	313.9	220.7	146.7
0207 51 90	Meat, not cut into pieces, fresh or chilled: plucked and drawn, without heads and feet (“75% geese”)	100.0	155.2	271.0	210.5	149.7	100.0	159.0	283.2	206.0	143.6
0207 52 90	Meat, not cut into pieces, frozen: plucked and drawn, without heads and feet (“75% geese”)	100.0	199.0	357.1	245.9	157.4	100.0	203.8	379.0	248.4	150.8
0207 55 10	Meat, in cuts, frozen, boneless	100.0	119.0	207.3	229.5	143.6	100.0	122.5	220.2	233.5	139.3
0207 55 31	Meat, in cuts, frozen, with bone: whole wings	100.0	74.9	79.6	99.0	133.4	100.0	77.4	84.9	102.1	130.5
0207 55 41	Meat, in cuts, frozen, with bone: backs, necks, rumps or wing-tips	100.0	214.2	337.0	332.9	185.0	100.0	221.0	358.2	341.5	178.1
0207 55 51	Meat, in cuts, frozen, with bone: breasts	100.0	171.0	346.3	251.7	153.8	100.0	175.2	367.9	254.9	147.2
0207 55 61	Meat, in cuts, frozen, with bone: legs	100.0	153.3	278.8	191.9	144.1	100.0	157.1	296.4	194.4	138.0
0207 55 81	Meat, in cuts, frozen, with bone: other than halves, quarters, whole wings, backs, necks, rumps, wing-tips, breasts, legs or paletots	100.0	48.0	89.4	65.2	123.5	100.0	50.3	96.9	68.1	122.3
0207 55 99	Edible offal, frozen: other than livers	100.0	86.2	87.2	109.8	131.4	100.0	89.5	93.1	113.9	128.8
Total	Imports	100.0	114.3	296.2	220.1	97.1	100.0	115.5	306.2	222.2	92.5
0207 55 51	Meat in cuts, frozen, breasts	100.0	---	156.9	125.9	---	100.0	---	167.6	127.0	---
0207 55 61	Meat in cuts, frozen, legs	100.0	88.3	235.4	---	52.5	100.0	89.5	251.7	---	51.0
0207 55 99	Edible offal, frozen, other than livers	100.0	119.0	228.0	156.4	---	100.0	124.2	243.6	160.1	---

Source: own development based on [23,24]. Prices in imports of other product groups are impossible to compare as imports occur too seldom (not every year).

## Data Availability

The original contributions presented in this study are included in the article. Further inquiries can be directed to the corresponding author.
